# Antioxidant effect of *Phyllanthus emblica* extract prevents contrast-induced acute kidney injury

**DOI:** 10.1186/1472-6882-14-138

**Published:** 2014-04-22

**Authors:** Adis Tasanarong, Supranee Kongkham, Arunporn Itharat

**Affiliations:** 1 Nephrology Unit, Department of Medicine, Faculty of Medicine, Thammasat University (Rangsit Campus), Klong Nung, Klong Luang, Pathumtani 12121, Thailand; 2 Department of Preclinical Sciences, Faculty of Medicine, Thammasat University (Rangsit Campus), Klong Nung, Klong Luang, Pathumtani 12121, Thailand; 3 Division of Applied Thai Traditional Medicine, Faculty of Medicine, Thammasat University (Rangsit Campus), Klong Nung, Klong Luang, Pathumtani 12121, Thailand

**Keywords:** *Phyllanthus emblica*, Gallic acid, Antioxidant, Contrast-induced acute kidney injury

## Abstract

**Background:**

Contrast-induced acute kidney injury (CI-AKI) occurs after the administration of intravenous iodinated contrast agents. Oxidative stress has been proposed as one of the most important mechanisms in the pathogenesis of CI-AKI. The objective of this study was to investigate the antioxidant effect of the extract from *Phyllanthus emblica* (PE) in preventing CI-AKI.

**Methods:**

Male Sprague Dawley rats were subjected into eight groups, were given water (control) or PE extract (125 or 250 or 500 mg/kg/day) for 5 days before the induction of CI-AKI. Renal function and oxidative stress markers; malondialdehyde (MDA), total antioxidant capacity (TAC), superoxide dismutase (SOD) and catalase (CAT) activity were determined in plasma and renal tissue. Kidney sections were performed for histopathological examination.

**Results:**

In the contrast media (CM) group, increases in blood urea nitrogen and serum creatinine were demonstrated which correlated with severity of tubular necrosis, peritubular capillary congestion and interstitial edema. Moreover, an increase in MDA and a decrease in TAC SOD and CAT activity in CM group were significantly changed when compared with the control (P < 0.05). In contrast, CI-AKI-induced rats administrated with PE extract 250 and 500 mg/kg/day significantly preserved renal function and attenuated the severity of pathological damage (P < 0.05) as well as significantly lower MDA and higher TAC, SOD and CAT than the CM group (P < 0.05).

**Conclusions:**

This study demonstrated the protective role of PE extract against CI-AKI.

## Background

Contrast-induced acute kidney injury (CI-AKI) is the common iatrogenic cause of acute kidney injury (AKI) following the use of intravenous contrast media (CM) [1,2]. The incidence of CI-AKI is 1 to 2% in low risk patients with normal renal function but increases up to 25% in high risk patients with chronic renal impairment or diabetes mellitus [2]. CI-AKI particularly in chronic kidney disease patients increases the hospitalizations and long term morbidity and mortality [3]. The pathogenesis of CI-AKI is due to the ability of contrast agents could induce renal vasoconstriction followed by hypoxic condition in the kidney which promotes further ischemic renal injury by the increase of oxygen free-radicals through oxidative stress [4]. Renal injury occurs when the hypoxic tissue generates reactive oxygen species (ROS) exceed than antioxidant reservation in the renal tissue [5]. Many strategies to prevent CI-AKI have been tested in many experimental studies and clinical trials. Hydration with isotonic saline has been recommended to prevent CI-AKI [6]. Additional antioxidants agents such as *N*-acetylcysteine, ascorbic acid and others demonstrated the conflicting results to CI-AKI prevention [7-9].

*Phyllanthus emblica* (PE) also known as Amla or Indian Gooseberry or Ma-Khaam-Pom in Thai is a natural fruit that contains high concentrations of acorbic acid [10,11], gallic acid [12], and mixture of phenolic compounds [13]. Active extracts of PE have been shown to posses anticancer [14,15], radioprotection [16], anti-inflammatory [17] and antioxidant [18,19] properties in several models of organ injury.

PE has never been used to assess its antioxidant effect in CI-AKI. The present study was designed to investigate the benefit of the PE extract in preventing CI-AKI and to discover the active substance in PE extract on CI-AKI prevention.

## Methods

### Sample

Phyllantus embelica fruits collected from Ampor Marim, Chiengmai Province Thailand on December 2011, were dried by oven at 50°C. Dry plant material (10 kg) was grinded and boiled in water for 30 min, filtered and evaporated by evaporator. The extract was dried by freeze dry as water extract of PE (PEW). The percentage of yield obtained as 40.81%. The samples have been preserved in the refrigerator (-20°C). Authentication of plant materials was identified by comparing against the specimens deposited at the herbarium of Southern Center of Thai Medicinal Plants, Faculty of Pharmaceutical Science, Prince of Songkla University, Songkla, Thailand, where herbarium vouchers have been kept (Herbarium no.SKP 071160501).

### Chemicals

Ascorbic acid, gallic acid and quercetin, thiobarbituric acid (TBA), 2, 2’–azinobis (3-ethylbenzothia-zoline-6-sulfonic acid) diammonium salt (ABTS), Trolox and all other chemical reagents were purchased from Sigma-Aldrich (Singapore). The low-osmolar, nonionic contrast-media agent (Iopromide) was obtained from Schering AG (Germany).

### Preparing standard ascorbic acid, Gallic acid and Quercetin

Ascorbic acid, Gallic acid and Quercetin were prepared as concentration as 1 mg/ml. Ascorbic acid and quercetin were prepared for 5 concentration per 1 millitre such as 6.25, 12.5, 25, 50 and 100 μg/ml. Gallic acid were also prepared for 5 concentration per 1 millitre such as 25, 50, 100, 200 and 400 μg/ml. Injection for these standard compounds for triplicate (N = 3).

### Preparing for sample

Preparing PEW as concentration 10 mg/ml was filtrated by syringe filter nylon, pore size 0.45 μm and injected in High Performance Liquid Chromatography (HPLC) instrument by triplicate (N = 3).

### HPLC analysis

The HPLC instrument is belong to Agilent Co. LTD (LC1200) (USA), Column: Phenomenex® Luna 5 μ C18(2) 100A size 250x4.60 mm 5 micron**,** Mobile phase: 0.1% Acetic acid in water (A): Methanol (B), gradient elution; 0–15 min as 5% B, 15–40 min as 80% B, 40–42 min as 5% B and 42–45 as 5% B**,** Flow rate: 0.9 ml/min**,** Detection: UV 280 nm**,** Injection volume: 20 μl.

### Animals

Male Sprague Dawley rats from the national animal center (Mahidol University) were used. The experiments were approved by the animal ethics committee of faculty of medicine, Thammasat University. All rats were housed in a temperature controlled room (24°C ± 1°C) and were given standard food pellets and tap water until they weighed 250-300 g, prior to the induction of CI-AKI. Rats were divided into eight groups of 6 rats to each group as follows: group 1, rats given sterile water (control); group 2, rats given sterile water followed by CI-AKI induction (CM); group 3, rats given PE extract 125 mg/kg/day (PE 125 mg); group 4, rats given PE extract 125 mg/kg/day followed by CM (PE 125 mg + CM), rats in both group 5 and group 6 given PE extract 250 mg/kg/day (PE 250 mg) but in group 6 was followed by CM (PE 250 mg + CM), group 7 and group 8 were given PE extract 500 mg/kg/day (PE 500 mg) but group 8 was followed by CM (PE 500 mg + CM). PE or sterile water were orally administrated for 5 days, and then rats in Group 2, Group 4, Group 6 and Group 8 were established with model of CI-AKI on the next day. The day after CI-AKI induction, all experimental animals were euthanized by inhalation of ether anesthesia.

### Introduction of CI-AKI in Rats

CI-AKI rats were subjected to CI-AKI protocol as described [20-22] briefly; pentobarbital sodium (60 mg/kg) anesthesia by intraperitoneal injection was followed by CI-AKI induction, which was performed with drug administration into tail vein. Drug administered consisted of indomethacin at a dose of 10 mg/kg, followed at 15 min and 30 min later with N^w^-nitro-L-Arginine methyl ester (L-NAME) at dose of 10 mg/kg and with low-osmolar, non-ionic contrast medium agent (Iopromide) at a dose of 1600 mg iodine/kg. This quantity is the dose of contrast medium that is standard for clinical use and for other relevant experiments in rat models. As controls, rats were injected with equivalent volume of saline at each time.

### Histopathologic examination of renal tissues

Both kidneys were excised immediately and cut into four equatorial sections; they were then washed twice with cold PBS. Two pieces of kidney were placed in 10% formaldehyde for histopathological examination. The other pieces were flash-frozen in liquid nitrogen, and stored at -70°C until used for subsequent tissue analysis. Histological slides of the formalin-maintained samples were prepared and counterstained with hematoxylin, eosin (H&E) and periodic acid schiff staining (PAS). These steps were then followed by semi-quantitative analysis the kidney section by a pathologist functioning in a blind manner [21,23,24]. Tubular necrosis and proteinaceous cast were graded according to a previous methodology as follows: 0 = no damage; 1 = mild (unicellular, patchy isolated damage); 2 = moderate (damage < 25%); 3 = severe (damage between 25 and 50%) and 4 = very severe (>50% damage). The degree of the medullary congestion was defined as: 0 = no congestion; 1 = mild (vascular congestion with identification erythrocytes by × 400 magnification); 2 = moderate (vascular congestion with identification of erythrocytes by × 200 magnification); 3 = severe (vascular congestion with identification of erythrocytes by × 100 magnifications) and 4 = very severe (vascular congestion with identification of erythrocytes by × 40 magnifications). Interstitial edema was graded as follows: 0 = no edema; 1 = mild (unicellular, patchy isolated edema); 2 = moderate (edema < 25%); 3 = severe (edema between 25 and 50%) and 4 = very severe (>50% edema).

### Biochemistry assay

In order to measure the oxidative stress markers including the oxidation of lipids, the antioxidant enzyme, total antioxidant capacity (TAC) and the renal function test, the serum was separated from blood that had been obtained from the heart and was kept at -20°C until used. Renal function was investigated in serum blood urea nitrogen (BUN), and serum creatinine levels were determined using an automatic analyzer. All measurements were performed using standard methods in a single, hospital-based laboratory [25]. Also, oxidative stress markers were measured in kidneys. Renal tissue samples were prepared from 0.1 g of frozen renal tissue which were homogenized in 1 ml of PBS (pH 7.2), and centrifuged at 10,000 g for 10 min at 4°C; supernatant was then taken and kept at -80°C until used. Protein contents in tissue homogenates were estimated by Bradford method using bovine serum albumin as a standard.

Lipid peroxidation, in term of malondialdehyde (MDA) was measured using thiobarbituric acid reactive substances (TBARS) assay, a method modified from that of Wong et al. [26]. Briefly, 100 ml of serum or renal tissue samples were subjected to 0.75 ml of phosphoric acid solution (0.44 M) and incubated at room temperature for 10 min; 5 ml of TBA solution was then added. The mixtures were heated at 100°C for 30 min. The samples were centrifuged at 4000 g for 10 min. The absorbance of the supernatant was read at 532 nm. The concentration of MDA was calculated from 0-20 μM of standard MDA using 1,1,3,3 tetraethoxypropane. The results of the serum or renal MDA were expressed as μM or μM per mg of protein, respectively.

Total antioxidant capacity (TAC) was determined by modified ABTS decolorization method as described by Re et al. [27] ABTS (7 mM in final concentration), with potassium per sulfate added (2.45 mM final concentration). The initial absorbance of induced ABTS radical cation solution was approximately 0.7-0.02 at 734 nm. After addition of 10 ml of sample or Trolox standard to 1 ml of diluted ABTS, the absorbance was measured at 734 nm after initial mixing. The TAC value was estimated by the reduction of absorbance at 734 nm and calculated against the curve of Trolox (0-1 μM). The values of TAC were expressed in equivalence of μM Trolox or Trolox/mg of protein, when measured in the serum or renal tissue samples, respectively.

Renal superoxide dismutase (SOD) activity was also determined using the SOD kit (Sigma), following the manufacturing protocol. The SOD activity was measured and expressed as units per mg protein.

Renal catalase (CAT) activity was measured according to the modified method of Aebi et al. [28]. This method measures the exponential disappearance of H_2_O_2_ (10 mM) at 240 nm for 2 min at room temperature. The CAT activity was expressed as units per milligram of protein (one unit of catalase is equal to 1 μmol of H_2_O_2_ decomposition/min).

### Statistical analysis

Results were present as the means ± SE. Student’s *t*-test (when two groups were considered) and one-way ANOVA were used to determine the significance of differences in multiple group comparisons using SPSS software. A *p*-value of less than 0.05 was considered to be as significance. The area under the curve (AUC) was calculated using the trapezoid rule. The correlation between two continuous variables was assessed using Person’s correlation coefficient and the coefficient of determination.

## Results

### HPLC analysis of PE

**Figure 1:**
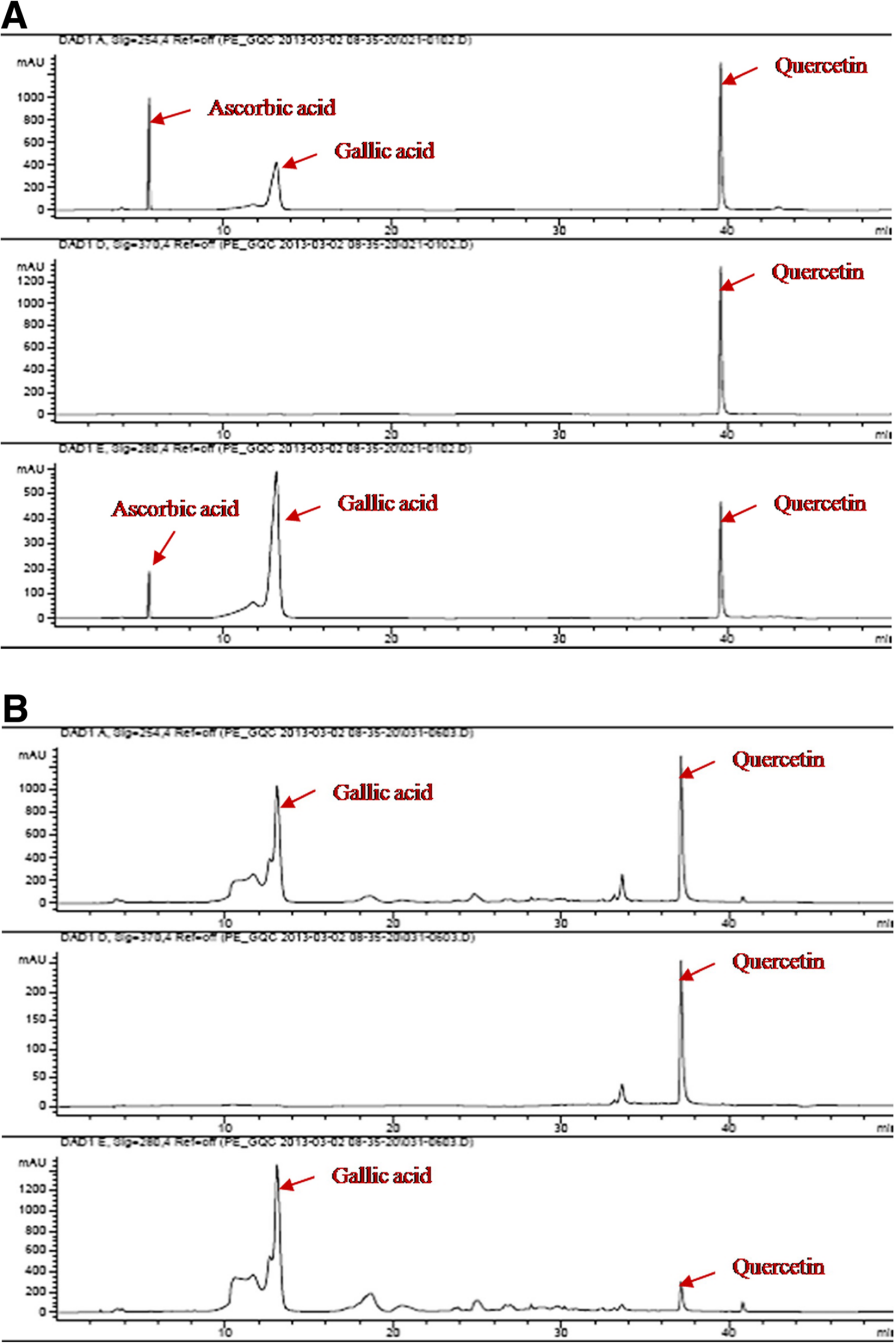
HPLC chromatogram of standard ascorbic acid, gallic acid and quercetin (1A) and PE extact (1B) at UV 254, 370 and 280 nm.

The five concentrations of ascorbic acid, gallic acid and quercetin were calculated and plotted as correlation graphs of concentration and AUC. The standard correlation between the concentration and AUC of ascorbic acid, gallic acid and quercetin were excellent, R^2^ = 0.999, 0.9998 and 0.9991 respectively. Figure 1A and B showed the HPLC chromatograms of standard ascorbic acid, gallic acid, quercetin and PE at UV 254, 370 and 280 nm. From HPLC analysis, the mean percentages of ascorbic acid, gallic acid and quercetin in PE extract by weight/weight were 0.00, 6.09 ± 0.38 and 0.68 ± 0.06 respectively.

### Effect of PE extract on renal function parameters

**Figure 2:**
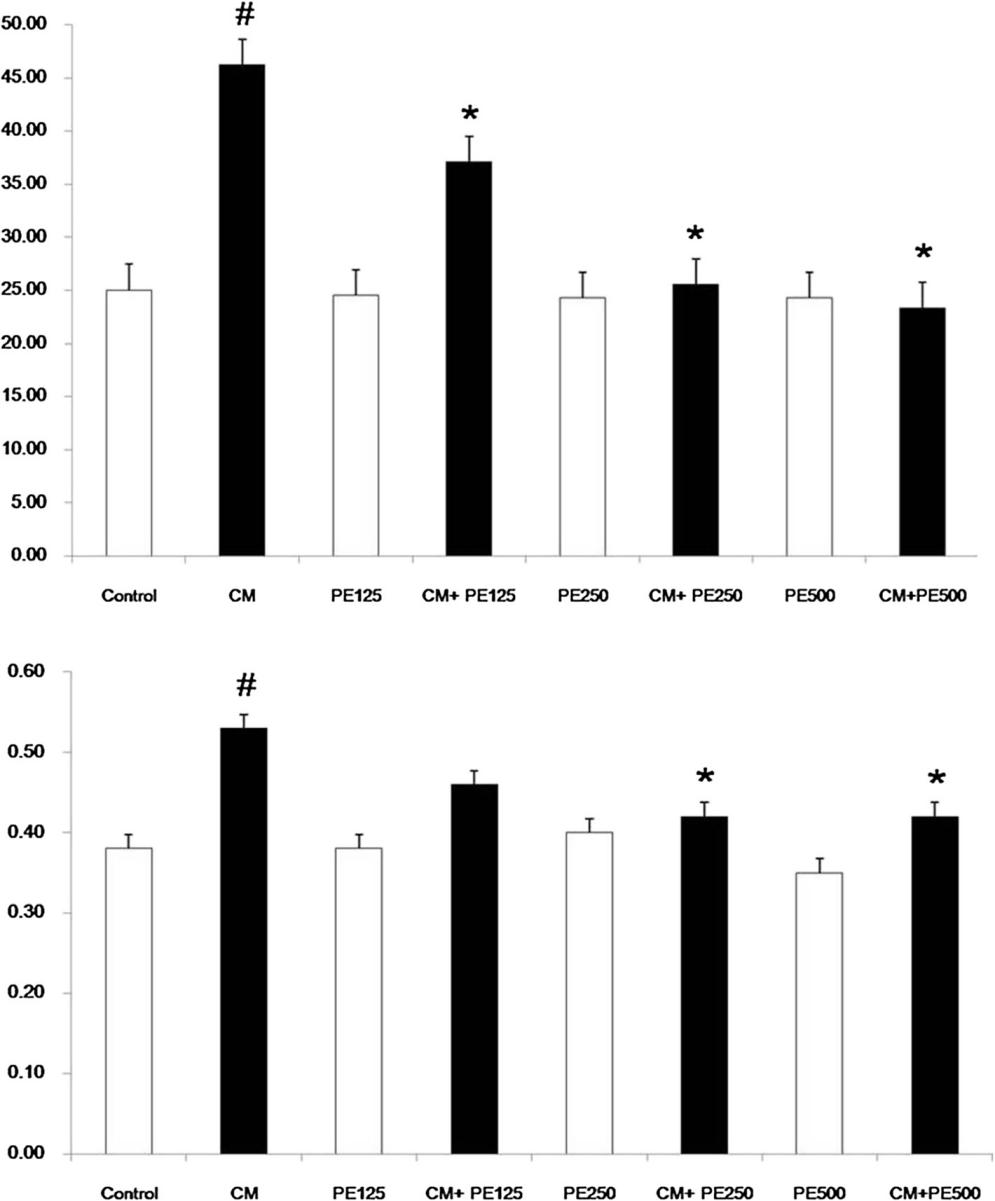
**Blood urea nitrogen and serum creatinine level in the eight experimental groups**. # P < 0.05; compared to control, * P < 0.05; compared to CM group.

Both BUN and serum Cr in the CM group were significantly increased when compared with the control (p < 0.05). While BUN was significantly decreased in the PE extract 125 mg + CM group, PE extract 250 mg + CM group and PE extract 500 mg + CM group when compared with CM group (P < 0.05); Figure 2. In addition, serum Cr was significantly decreased in only two of the PE extract 250 mg + CM group and PE extract 500 mg + CM group when compared with CM group (P < 0.05); Figure 2.

### Effect of PE extract on attenuation of renal histopathology

**Figure 3:**
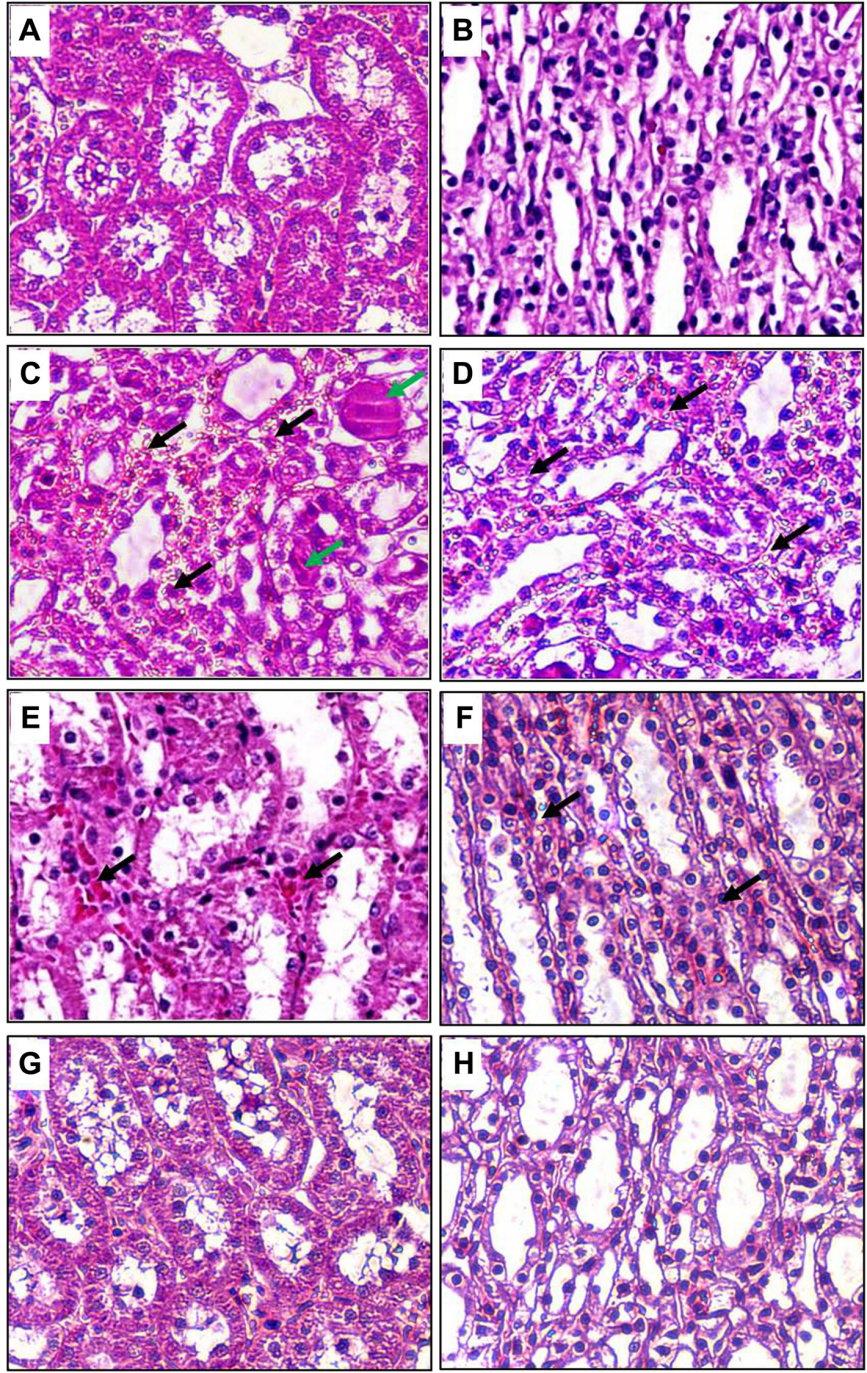
**Histopathological with H& E staining of CI-AKI in rats. A** and **B** show normal rat kidneys in the cortex and medulla. Rats induced with CM **(C and D)** demonstrate severe TEC necrosis, moderate PTC congestion (black arrows) and proteinaceous casts (green arrows) in the cortex and medulla. Rats induced with CM and pre-treatment with PE extract 250 mg/kg/day **(E and F)** show a decrease in the severity of TEC necrosis, PTC congestion, and proteinaceous casts in the cortex and medulla. Interestingly, rats induced with CM and pre-treatment with PE extract 500 mg/kg/day **(G and H)** demonstrate minimal TEC necrosis, PTC congestion or proteinaceous casts in the cortex and medulla, similar to control-rat kidneys. (Magnifications: ×400 in A-H). CM: contrast media; PTC: peritubular capillaries; TEC: tubular epithelial cells.

Histological evaluation of kidney sections in the CM group revealed the significantly severe damage consisting of tubular necrosis, proteinaceous cast formation, PTC congestion and interstitial edema in the outer stripe of the outer medulla (OSOM) and cortex when compared with the control (P < 0.05); Table 1 and Figure 3. Pretreatment with PE extract 250 mg and PE extract 500 mg in rats with CI-AKI induction were significantly attenuated the development of these lesions when compared with the CM group (P < 0.05); Table 1 and Figure 3.

**Table 1:** Histopathological analysis of tubular necrosis, PTC congestion and interstitial edema in the five experimental groups

	**Control (n = 6)**	**CM (n = 6)**	**CM PE 125 mg (n = 6)**	**CM PE 250 mg (n = 6)**	**CM PE 500 mg (n = 6)**
Tubular necrosis and proteinaceous casts	0.1 ± 0.3	2.1 ± 0.5^#^	1.6 ± 0.5	1.3 ± 0.5*	0.7 ± 0.5*
PTC congestion	0.3 ± 0.5	2.3 ± 0.5^#^	2.1 ± 0.5	1.8 ± 0.4*	0.8 ± 0.4*
Interstitial edema and infiltration	0.2 ± 0.4	2.4 ± 0.5^#^	2.1 ± 0.5	1.8 ± 0.4*	0.8 ± 0.4*

The score of histopathological changes, such as scores of tubular necrosis and proteinaceous cast, PTC congestion, interstitial edema and infiltration, are presented as means ± SD.

# P < 0.05; compared to control.

*P < 0.05; compared to CM.

CM: contrast media; PTC: peritubular capillaries.

### Effect of PE extract on attenuation of oxidative stress on CI-AKI

**Figure 4:**
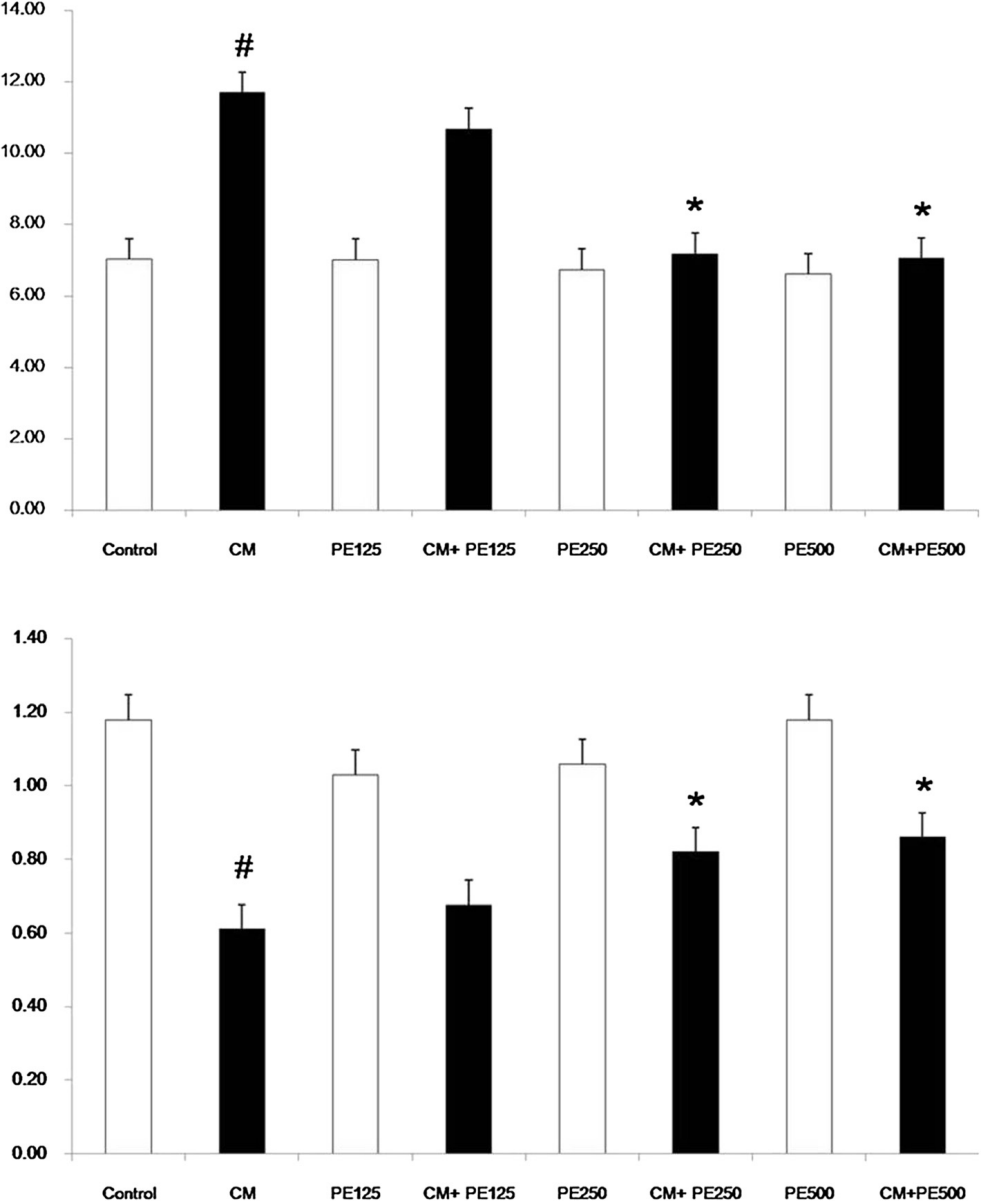
**Plasma MDA and TAC level in the eight experimental groups.** # P < 0.05; compared to control, *P < 0.05; compared to CM group.

**Figure 5:**
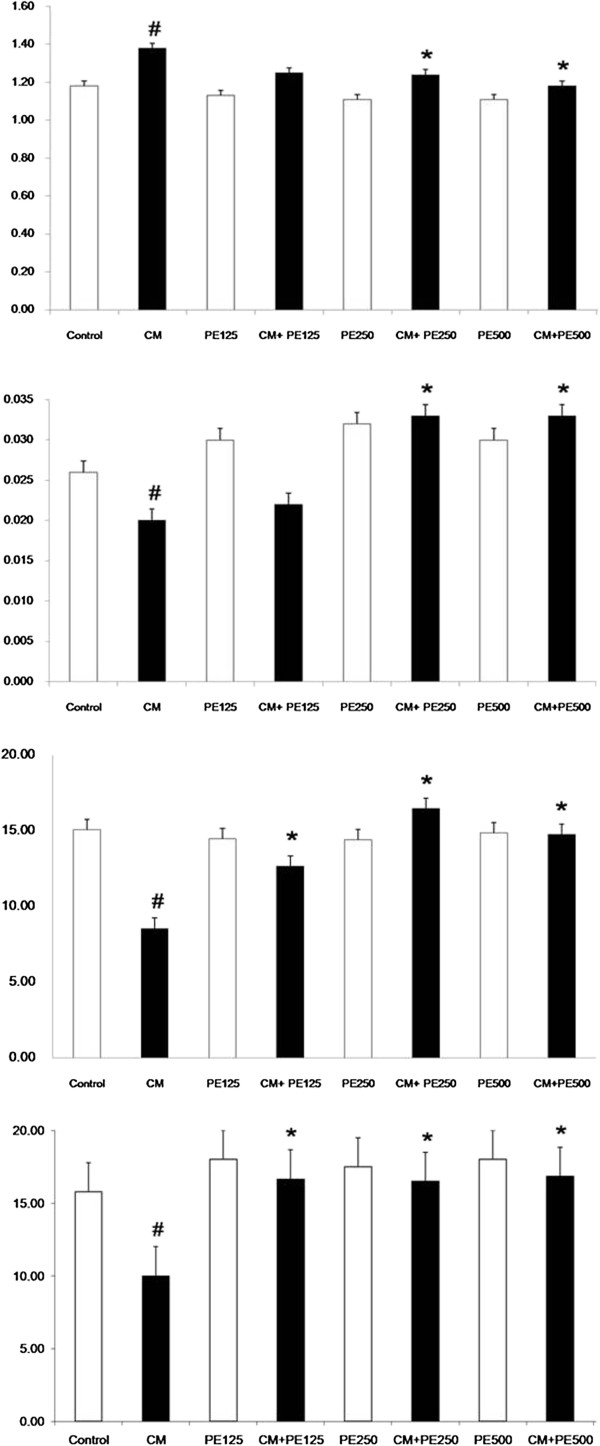
**Renal tissue MDA, TAC, SOD and CAT level in the eight experimental groups**. # P < 0.05; compared to control, *P < 0.05; compared to CM group.

The serum and renal MDA levels in the CM group were significantly increased when compared with the control (P < 0.05), whereas the MDA levels were significantly lower in the PE extract 250 mg + CM group and PE extract 500 mg + CM when compared with CM group (P < 0.05); Figures 4 and 5. The serum and renal TAC levels in the CM group were significantly decreased when compared with the control (P < 0.05), while the TAC levels in the PE extract 250 mg + CM group and PE extract 500 mg + CM group were significantly higher than the CM group (P < 0.05); Figures 4 and 5. Moreover the renal SOD and CAT levels were significantly decreased in CM group when compared with the control (P < 0.05); Figure 5. The renal SOD and CAT levels in the PE extract 125 mg + CM, PE extract 250 mg + CM and PE extract 500 mg + CM group were significantly higher than the CM group (P < 0.05); Figure 5.

## Discussion

To our knowledge, this is the first study to demonstrate the renoprotective effect of PE extract to prevent the development of renal dysfunction and pathological destruction as assessed by biochemical and markers of oxidative stress in a rat model of CI-AKI. Prophylaxis with PE extract at doses of 250 and 500 mg/kg/day markedly suppressed renal tubular injuries and improved antioxidant activity in CI-AKI rats.

The nephrotoxic effects of radiocontrast media have been reported in many experimental studies [21,24,29] and we followed the same methodology as reported elsewhere [20,21,30]. The histopathological studies confirmed significant acute tubular necrosis, vacuolization, loss of brush borders, proteinaceous cast formation and interstitial edema were demonstrated similar to previous studies in this nephropathy model [20,21,24,30]. We also found that serum BUN and Cr levels increased significantly in the CM group compared with the control group, supporting the nephrotoxicity of radiocontrast media to renal function. Pretreatment with PE extract demonstrated clearly its renoprotetctive effect by attenuating the severity of renal pathological damage and improving renal function with the dose dependent renoprotective effect of PE extract.

Oxidative stress has been proposed as one of the most important mechanisms in the pathogenesis of CI-AKI [31]. In vitro and in vivo studies demonstrate clearly that iodinated contrast media administration enhances hypoxia and increases the production of ROS within the kidney [21,24,32]. Lipid peroxidation is initiated as a result of the ROS induced abstraction of hydrogen in cellular membranes, which results in the formation of relatively stable compounds such as MDA. The production of free radicals is blocked by endogenous antioxidant systems such as TAC, SOD and CAT enzymes [21,24]. Thus, oxidative stress induced by contrast media during AKI is a relative excess of oxidants caused by increased free radical production and/or decreased antioxidant defense systems [20,21,24]. In the present study, increased MDA and decreased TAC, SOD and CAT enzyme activities in serum and renal tissues exposed to CM suggests that these enzymes were consumed due to increased oxidative stress. Decreased SOD and CAT scavenger activities results in higher H_2_O_2_ concentration and explains the change in the level of MDA during CI-AKI. The higher of antioxidant enzyme SOD and CAT activities and lower MDA in the PE extract pretreatment might be a response for renal injury after contrast administration. Thus, rats that receive PE extract before CM had decreased MDA and increased SOD and CAT activities in renal tissues could protected against CI-AKI from ROS. Moreover, the increased MDA and decreased TAC in rat plasma that exposed to CM reflect an increase in lipid peroxidation and decrease antioxidation in the systemic response. Thus, decreasing oxidative stress injury following PE extract application has been associated with antioxidant effects in circulation system.

Current recommendations to decrease the incidence of CI-AKI in high risk patients are hydration with saline, use of iso-osmolar contrast agents, minimum volume of contrast media, and avoiding nephrotoxic drugs [6,33]. Recent studies try to use antioxidants as protective remedies against CI-AKI have yielded conflicting results [34-36]. Antioxidant effect of PE was demonstrated in vitro and vivo studies by increased the concentration of antioxidant enzymes SOD and CAT and decreased MDA in lipid peroxidation [37-39]. The present study demonstrated that antioxidant effect of PE extract could decrease MDA in both plasma and renal tissues. Moreover, PE extract preserved plasma TAC and renal tissues TAC, SOD and CAT activities. These effects correlated with the attenuation of histopathological injury from contrast media administration. The dose dependent effect of PE extract started at dose 250 mg/kg/d and had the additional effect at dose 500 mg/kg/d similar to the antioxidant effect of vitamin E in the experimental study [21] and clinical trials [25,40]. Thus, the renoprotective effect of PE extract could prevent CI-AKI through antioxidant property.

*Phyllanthus emblica* is widely used in Thai traditional medicine and Ayurvedic Medicine for treatment of various diseases. The fruit of PE is known as a rich source of ascorbic acid [10,11], gallic acid [12], and also contains a mixture of phenolic compounds [13]. The major substrate which acts to be antioxidant in PE is gallic acid, vitamin C and others depending on the technique that separates the compound from PE [41,42]. From the process to prepared PE extract in the present study, plant was dried by oven at 50°C before grinded and boiled in water. In general, vitamin C was destroyed by heat that could be loss from the preparing process [43]. In addition, PE extract by HPLC analysis in the present study showed the main substrate was gallic acid. Gallic acid is a polyphenolic compound with multiple hydroxyl groups which are able to donate its proton to break the chain reaction of free radicals consequently as a lipid peroxidation inhibitor [44,45]. Moreover, gallic acid was demonstrated as an excellent antioxidant with high free radical scavenging effect [45,46]. From the experimental studies, gallic acid was demonstrated to prevent or attenuate the severity of brain, heart and liver damage from ischemic or toxic substances injury [47-49]. Furthermore, the reno-protective effect of gallic acid from antioxidant and antiinflammation properties was demonstrated in many experimental studies in AKI against lindane [49], ferric nitriloacetic acid [50], and sodium fluoride [47] and chronic kidney disease [51]. By our HPLC analysis, gallic acid was present in 6% of the PE extract, corresponding to doses of 15 and 30 mg/kg/d in the 250 and 500 mg/kg/d of PE extracts, respectively. These doses of gallic acid could be the basis of human studies. Thus PE extract administration could prevent CI-AKI in rats with antioxidant effect from gallic acid. These finding could be assessed in patients with high risk CI-AKI in the future clinical trials.

The plasma levels of PE extract and the accumulation of PE extract on renal tissues with the repeated daily dose may be higher than the single dose administration. In experimental study, the bioavailability of gallic acid from grape seed polyphenol extract is improved by repeated dosing in rats [52]. The single dose with oral administration demonstrated that the intestinal absorption of gallic acid is poor (<2%). While repeated exposure to the extract has shown the absorption of gallic acid was significantly higher than single dose treatment and reached to tissues level at day 10. Moreover, two gallic acid-derived compounds isolated from Casearia sylvestris leaves could reverse NK cell cytolysis which was suppressed from tumor growth when mice were treated with these compounds for 4 days [53]. In contrast, a single dose administration with a large volume and high concentration of any compound via oral route may cause adverse effects and could not reach to the therapeutic levels in the tissues. From these informations, we make the decision to give PE extract orally as presented in the protocol. However, a high dose administration of PE extract for 2 or 3 days before contrast administration should be evaluated for the preventing CI-AKI.

### Study limitations

Monitoring of urine output and/or novel biomarker in plasma or urine is a much better indicator for early diagnosis of CI-AKI. However, we have measured only serum BUN and Cr levels. Also, we have only H&E and PAS staining for histopathologic examination of renal tissues. Therefore, the immunohistochemistry staining such as Tunnel or PCNA and the evaluation of protein and gene expression in apoptosis or inflammatory pathway should be prepared to confirm CI-AKI.

## Conclusion

The present study suggests that PE extract administration pretreatment for five days in dose 250 and 500 mg/kg/day before the induction of CI-AKI exerts significant renoprotective effects in a rat model of CI-AKI. These finding indicate that PE extract could represent a novel and effective preventive approach for CI-AKI as a result of its antioxidant capacity to preserve renal function and directly protect renal tissues. Investigation with additional experimental studies and clinical trials is required to confirm to the advantage of PE extract to prevent the CI-AKI.

## Abbreviations

CI-AKI: Contrast-induced acute kidney injury; AKI: Acute kidney injury; CM: Contrast media; ROS: Reactive oxygen species; PE: *Phyllanthus emblica*; HPLC: High Performance Liquid Chromatography; MDA: Malondialdehyde; TAC: Total antioxidant capacity; SOD: Superoxide dismutase; CAT: Catalase; BUN: Blood urea nitrogen; AUC: Area under the curve.

## Competing interests

All authors declare that they have no competing interest.

## Authors’ contributions

AT conceived the idea of study, designed the study protocol, collected the data, interpreted the data and wrote the manuscript. SK participated in the conceptualization of the design, carried out the technical aspect of the study, collected the data and revised the manuscript. AI prepared the samples, carried out the HPLC aspect of the study, collected the data and revised the manuscript. All authors read and approved the final manuscript.

## Pre-publication history

The pre-publication history for this paper can be accessed here:

http://www.biomedcentral.com/1472-6882/14/138/prepub
